# Immunohistochemical Expression of TNFR1, IL-6, and TGF-β1 in the Synovial Tissue of Patients with Hip Osteoarthritis

**DOI:** 10.3390/biomedicines13112732

**Published:** 2025-11-07

**Authors:** Petar Todorović, Ivana Jurić, Nela Kelam, Matko Rošin, Davor Čarić, Danica Boban, Andrea Kopilaš, Katarina Vukojević

**Affiliations:** 1 Department of Anatomy, Histology and Embryology, School of Medicine, University of Split, 21000 Split, Croatia; petar.todorovic@mefst.hr (P.T.); nela.kelam@mefst.hr (N.K.); danica.boban@mefst.hr (D.B.); 2 Department of Emergency Medicine, University Hospital of Split, Spinciceva 1, 21000 Split, Croatia; ivana.juric@mefst.hr; 3 Surgery Department, Orthopaedics and Traumatology Division, University Hospital of Split, Spinciceva 1, 21000 Split, Croatia; mrosin@kbsplit.hr (M.R.); dcaric@kbsplit.hr (D.Č.); 4 Department of Family Medicine, Health Center Mostar, Bulevar Hrvatskih Branitelja b.b., 88000 Mostar, Bosnia and Herzegovina; andrea.kopilas@mef.sum.ba; 5 Center for Translational Research in Biomedicine, University of Split School of Medicine, Soltanska 2, 21000 Split, Croatia; 6 Mediterranean Institute for Life Sciences, University of Split, Meštrovićevo Šetalište 45, 21000 Split, Croatia

**Keywords:** hip osteoarthritis (HOA), synovial membrane, tumor necrosis factor receptor 1 (TNFR1), interleukin-6 (IL-6), transforming growth factor beta 1 (TGF-β1), Immunofluorescence

## Abstract

**Background/Objectives**: Hip osteoarthritis (HOA) is a progressive joint disease characterized by cartilage loss, subchondral bone changes, and synovial inflammation. While tumor necrosis factor receptor 1 (TNFR1), interleukin-6 (IL-6), and transforming growth factor-beta 1 (TGF-β1) are recognized as key mediators of joint pathology, their compartment-specific expression in the human hip synovium remains insufficiently characterized. Therefore, we aimed to investigate their localization and expression in the intimal and subintimal compartments of synovial tissue in patients with HOA compared to controls (CTRL). **Methods**: Synovial membrane samples were obtained from 19 patients with primary HOA undergoing total hip arthroplasty and 10 CTRL subjects undergoing arthroplasty for acute femoral neck fracture without HOA. Specimens were processed for hematoxylin and eosin (H&E) and immunofluorescence staining. Expression of TNFR1, IL-6, and TGF-β1 was quantified in the intima and subintima using ImageJ analysis. Group differences were assessed using two-way Analysis of variance (ANOVA) with Tukey’s test when assumptions were met; for heteroscedastic outcomes we applied Brown–Forsythe ANOVA with Dunnett’s T3 multiple comparisons. **Results**: Histological analysis confirmed synovitis in HOA samples, with intimal hyperplasia and mononuclear infiltration. IL-6 was significantly upregulated in the intima of HOA synovium compared with CTRLs, while subintimal expression remained unchanged. In contrast, TGF-β1 expression was reduced in the HOA intima, eliminating the normal intima–subintima gradient. For TNFR1, the within-HOA contrast (int > sub) was significant, whereas the intimal HOA vs. CTRL comparison showed a non-significant trend. Transcriptomic analysis supported IL-6 upregulation, while TNFR1 and TGF-β1 did not reach statistical significance at the mRNA level in an orthogonal, non-hip (knee-predominant) dataset. **Conclusions**: These findings demonstrate compartment-specific cytokine dysregulation in HOA, with increased intimal TNFR1 and IL-6 alongside reduced intimal TGF-β1. The synovial lining emerges as a dominant site of inflammatory signaling, underscoring its importance in disease progression.

## 1. Introduction

Osteoarthritis (OA) is a degenerative joint disorder characterized by progressive articular cartilage breakdown, subchondral bone remodeling, osteophyte formation, synovial inflammation, and periarticular muscle weakening [1]. These pathological changes result in pain, stiffness, and impaired mobility, which substantially reduce quality of life and increase the risk of disability [2]. Globally, OA affects about 7.6% of the population, with prevalence projected to rise dramatically by 2050, making it one of the leading causes of disability in individuals over 70 years [3]. While the knee is most frequently affected, the burden of hip osteoarthritis (HOA) has increased by more than 120% between 1990 and 2019, underscoring its clinical significance [4].

Established risk factors for HOA include age, female sex, obesity, previous joint injury, heavy occupational load, metabolic disorders such as diabetes, and abnormal hip morphology (dysplasia or femoroacetabular impingement) [5,6]. Traditionally considered a wear-and-tear condition, OA is now recognized as a multifactorial disease driven by mechanical, metabolic, and inflammatory processes that affect the entire joint [2]. Synovial inflammation (synovitis) is now recognized as a central feature of OA progression. Characterized by immune cell infiltration, synovial lining hyperplasia, and elevated pro-inflammatory cytokines, synovitis can precede cartilage loss and strongly correlates with pain and structural deterioration in OA [7,8,9]. Proinflammatory cytokines, particularly IL-1β and IL-6, as well as signaling via tumor necrosis factor receptor 1 (TNFR1), are considered major mediators of this process, orchestrating cartilage catabolism, matrix metalloproteinase release, and amplification of the inflammatory cascade [9,10].

Among the cytokines involved, TNFR1 (CD120a), interleukin-6 (IL-6), and transforming growth factor-beta 1 (TGF-β1) are key mediators of inflammation and tissue remodeling in OA [7,8]. TNFR1 is the primary receptor through which TNF-α exerts its catabolic effects, activating the classical NF-κB pathway and driving transcription of pro-inflammatory genes, chemokines, and matrix-degrading enzymes [10,11,12,13]. In OA, persistent TNFR1-mediated signaling amplifies synovial inflammation and promotes cartilage degradation, a process similar to that observed in other chronic inflammatory conditions, such as rheumatoid arthritis (RA) and psoriasis [13].

IL-6 is a pleiotropic cytokine that regulates immune responses, tissue homeostasis, and acute-phase reactions [14]. It is produced by synovial fibroblasts, lymphocytes, and other joint-resident cells, and is consistently elevated in OA synovial fluid [7,14]. IL-6 signals via the JAK/STAT pathway, inducing transcription of genes involved in inflammation, angiogenesis, and cell proliferation [14,15]. Although essential in acute defense, sustained IL-6 expression drives chronic inflammation, promotes osteoclastogenesis, and contributes to fibrosis and angiogenesis, thereby accelerating joint damage [16,17]. Dysregulated IL-6 signaling, often downstream of NF-κB activation, is a recognized hallmark of OA and other chronic inflammatory diseases [16].

In contrast, TGF-β1 plays a dual role in joint biology. Under physiological conditions, it maintains cartilage integrity by stimulating extracellular matrix (ECM) synthesis and suppressing inflammation [18]. TGF-β1 is stored in the ECM in a latent form and activated by injury, mechanical loading, or enzymatic cleavage [18]. Its canonical SMAD2/3 signaling exerts protective effects, while non-canonical pathways such as MAPK further regulate cell survival and ECM remodeling [18,19]. However, chronic elevation of TGF-β1 in OA skews signaling toward the SMAD1/5/8 axis, leading to cartilage hypertrophy, osteophyte formation, subchondral bone changes, and synovial fibrosis [20,21,22]. This shift from protective to pathological activity highlights its context-dependent role in OA pathogenesis.

Given their central yet interrelated roles, examining the expression of TNFR1, IL-6, and TGF-β1 in the synovium is crucial for understanding the molecular mechanisms of HOA. While each cytokine contributes distinctly to inflammation and remodeling, their pathways intersect and modulate one another within a complex network of signaling. However, their combined expression patterns in HOA synovium remain poorly defined. Because viable, non-osteoarthritic hip synovial tissue suitable for molecular manipulation or in vitro functional assays cannot be ethically or technically obtained, a descriptive human-tissue approach remains the only feasible design for this setting. Accordingly, this study was conceived as an observational, cross-sectional analysis aimed at mapping compartment-specific expression of TNFR1, IL-6, and TGF-β1 in human hip synovium. While not designed for functional validation, it provides a translational morphological baseline that can guide and contextualize future mechanistic investigations. 

## 2. Materials and Methods

### 2.1. Study Population

The Ethics Committee of University Hospital Split in Split, Croatia, approved the research (protocol code: 500-03/23-01/230; date of approval: 27 November 2023). The research was conducted following the rules of the Declaration of Helsinki. In the clinical part of the research, which was carried out at the Department of Orthopedics and Traumatology of University Hospital in Split, we performed the selection of subjects and the surgical procedures, in which we sampled hip tissue, while the tissue processing and staining of the samples were carried out at the Department of Anatomy, Histology and Embryology, University of Split School of Medicine. All respondents voluntarily decided to participate in the research and signed their consent to participate. This investigation was designed as an observational, cross-sectional study based on human surgical specimens. Because viable, non-osteoarthritic hip synovium suitable for in vitro manipulation or cytokine stimulation cannot be ethically or technically obtained, functional assays were not feasible; therefore, the study focused on morphological and compartment-specific expression mapping of key cytokines. In all participants, synovial tissue was obtained from the same hip joint and, to ensure consistent anatomical sampling, from the anterior–superior capsular synovial region adjacent to the femoral neck.

This study included a total of 29 participants, comprising 19 patients with primary hip osteoarthritis (HOA) who underwent total hip arthroplasty and 10 control (CTRL) subjects who underwent hip arthroplasty due to an acute femoral neck fracture, without radiological or clinical evidence of HOA. The clinical, radiological, and pathohistological characteristics of both groups are summarized in Table 1.

All HOA patients fulfilled the clinical and radiographic criteria for advanced disease, including persistent hip pain, reduced joint function, and a Kellgren–Lawrence grade ≥ 3 [23]. Conservative treatment had been attempted in all cases prior to surgery, but joint function remained limited, and pain was long-standing. Hip arthroplasty was performed through a posterolateral surgical approach, involving incision of the short external rotators and posterior capsule, followed by joint luxation. From each HOA patient, the most damaged cartilage zone in the weight-bearing area of the femoral head was removed in a triangular block using an oscillating saw (Trauma Recon System, Synthes, Switzerland) and processed for histological and immunofluorescence analysis. HOA patients were not receiving chronic systemic corticosteroids or immunosuppressive therapy at the time of surgery.

Control subjects were patients undergoing arthroplasty for recent femoral neck fracture, with no prior hip symptoms, no radiographic signs of HOA (Kellgren–Lawrence grade 0–1), and no history of rheumatological or infectious hip disease. To further exclude inflammatory arthritis, individuals with positive anti-cyclic citrullinated peptide antibodies (anti-CCP) or rheumatoid factor (RF) were not included. In all CTRL cases, synovial tissue was collected during the index arthroplasty within 48 h of injury to minimize secondary inflammatory remodeling. CTRL patients had no systemic inflammatory or rheumatologic conditions, were not on chronic corticosteroid or immunosuppressive therapy, and their synovial membranes showed no histological evidence of synovitis (Krenn score = 0). Although acute trauma can elicit a short-lived local response, prior reports indicate that such transient changes remain minimal compared with the chronic inflammatory activation characteristic of HOA; therefore, this cohort provides an ethically accessible and valid baseline for evaluating disease-specific alterations in cytokine expression.

Functional status and pain intensity in the HOA group were assessed preoperatively using the Harris Hip Score (HHS) [24], the Western Ontario and McMaster Universities Osteoarthritis Index (WOMAC) [25], and a visual analogue scale (VAS) [26]. All three evaluations were performed during the same preoperative visit by the same trained orthopedic assessor to ensure consistency and minimize inter-observer variability. Histopathological evaluation was performed in both groups using the Krenn synovitis score, which allowed confirmation of the synovial inflammation status [27].

Exclusion criteria for both groups included a history of inflammatory rheumatic disease, previous hip infection, secondary causes of HOA (such as developmental dysplasia or femoroacetabular impingement), metabolic bone disease, or malignancy. Patients on chronic systemic corticosteroid or immunosuppressive therapy were excluded from both cohorts. In addition, any intra-articular corticosteroid injection within the preceding 3 months was an exclusion criterion to avoid transient modulation of synovial cytokine levels.

### 2.2. Tissue Collection and Basic Staining Procedures

During total hip arthroplasty, synovial membrane specimens were collected from the anterosuperior portion of the joint capsule adjacent to the femoral head, representing the weight-bearing region. Samples were immediately fixed in 10% neutral buffered formalin for 24 h, dehydrated through graded alcohols, cleared in xylene, and embedded in paraffin. Paraffin blocks were sectioned at 5 µm thickness using a rotary microtome (Leica, Wetzlar, Germany).

**Figure 1:**
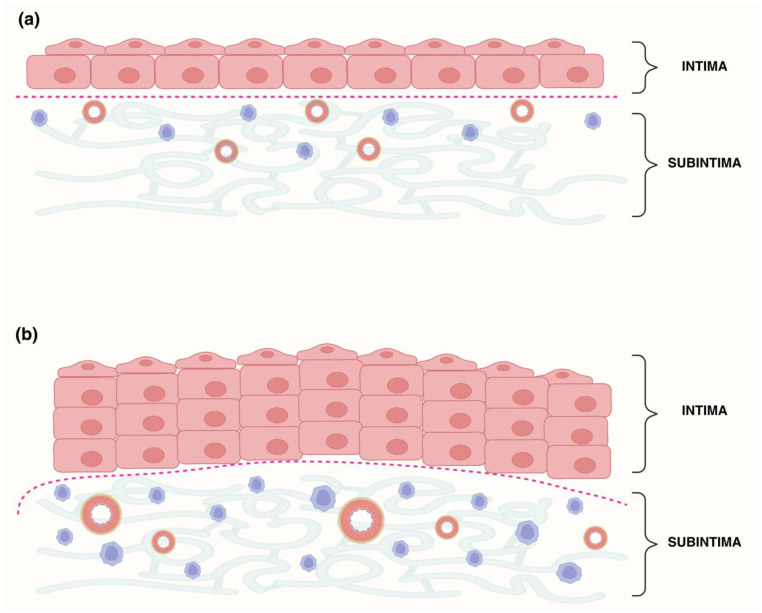
Schematic of synovial layer delineation in control (Ctrl) and hip osteoarthritis (HOA) samples. (**a**) Ctrl synovial membrane with a thin intima (1–3 fibroblast-like synoviocyte layers) overlying a subintima with sparse stroma, few vessels, and low cellularity. (**b**) HOA synovial membrane showing intimal hyperplasia and an expanded subintima enriched in blood vessels and inflammatory cells. The red dotted line indicates the manually defined intima–subintima boundary, traced with the Lasso tool in Adobe Photoshop based on morphology.

For routine histological evaluation, sections were stained with hematoxylin and eosin (H&E) following standard protocols. Stained slides were examined under a light microscope (Olympus BX series, Tokyo, Japan) to verify tissue morphology and preservation of synovial architecture. Only well-preserved specimens containing both intimal and subintimal layers were included in the subsequent immunofluorescence analyses. For compartment definition, the intima was identified as the continuous synoviocyte lining at the tissue surface (avascular, without adipocytes, whereas the subintima was defined as the underlying fibro-areolar connective tissue containing microvessels, nerves, collagen bundles, and occasional adipocytes. The intima–subintima boundary followed the deep margin of the most basal, contiguous lining cells; in villous projections, the entire uninterrupted surface lining was considered intima. Delineation was performed by an experienced histologist and a board-certified pathologist, blinded to group assignment, with consensus in case of discrepancy. A schematic illustrating the histological criteria and boundary tracing used to separate intima from subintima is shown in Figure 1.

### 2.3. Immunofluorescence Staining

Paraffin-embedded synovial sections (5 µm) were deparaffinized in xylene and rehydrated through a descending ethanol series. Antigen retrieval was performed by heating slides in 0.01 M citrate buffer (pH 6.0) at 95 °C for 30 min in a water steamer, followed by gradual cooling to room temperature. After rinsing in 0.1 M PBS, nonspecific binding was blocked using a commercial protein-blocking reagent (ab64226, Abcam, Cambridge, UK) for 20 min.

The sections were incubated overnight at 4 °C in a humidity chamber with primary antibodies against TNFR1/CD120a, IL-6, and TGF-β1 (Table 2). The following day, slides were washed with PBS and exposed for 1 h to the appropriate secondary antibodies (Table 2). After further PBS rinses, nuclei were counterstained with 4′,6-diamidino-2-phenylindole (DAPI), and sections were mounted with Immu-Mount (Thermo Shandon, Pittsburgh, PA, USA).

Standard negative controls confirmed antibody specificity: (i) omission of the primary antibody, which resulted in no detectable staining, and (ii) preadsorption with the corresponding blocking peptide when available, which abolished the immunoreactive signal. The staining patterns observed in positive controls were consistent with the expected cellular localization reported in the literature, further supporting antibody specificity.

### 2.4. Data Acquisition and Quantitative Analysis

The slides were examined with a fluorescence microscope (Olympus BX61, Tokyo, Japan) equipped with a Nikon DS-Ri2 camera (Nikon Corporation, Tokyo, Japan) and NIS-Elements F software, version 5.22.00, which was used to capture microphotographs. For each specimen, ten non-overlapping fields were taken at 40× objective magnification, encompassing both the intimal and subintimal layers of the synovium. Fields were selected where the surface lining was continuous and the boundary to the subintima was morphologically clear.

To enable quantitative analysis of immunoreactivity, the intimal compartment was separated from the subintima using the Lasso tool in Adobe Photoshop (v21.0.2, Adobe Systems, San Jose, CA, USA). The compartment masks were defined on H&E/IF morphology as described above (Figure 1); DAPI was used only as an orientation aid for nuclei and not for boundary determination. To minimize transition-zone misclassification, a narrow exclusion margin was applied along the traced boundary before measurement. Images were then processed with ImageJ software, version 1.54 (NIH, Bethesda, MD, USA) to isolate the positive immunostaining signal, as previously described [28]. The workflow included duplicating each image, separating the red, green, and blue channels, and subtracting the red channel from the original image to reduce the background. The processed images were further filtered using a median filter (radius 10.0 pixels) and the “image calculator” function in ImageJ. Thresholding was applied using the triangle method, and the “analyze particles” tool was used to calculate the area percentage occupied by positive staining.

Because parts of the images contained an empty background without tissue, a correction was applied to avoid underestimation of staining. Using the ”Magic Wand” tool in Photoshop, the total number of pixels and the number of pixels representing empty space were determined. The corrected area percentage was calculated by multiplying the uncorrected value by the ratio of total pixels minus background pixels to the total pixel count, as described previously [28,29]. 

### 2.5. Differential Gene Expression

Publicly available gene expression data were retrieved from the Gene Expression Omnibus (GEO) database of the National Center for Biotechnology Information (NCBI) [30]. A search using the keywords “*osteoarthritis*,” “*Homo sapiens*,” and “*Expression profiling by array*” yielded 114 datasets. From these, we selected the GSE55235 series (*Rheumatoid arthritis and osteoarthritis: synovial tissues, Berlin dataset*), which includes expression profiles from 30 synovial tissue samples: 10 from patients with osteoarthritis (OA), 10 from rheumatoid arthritis (RA), and 10 from age- and sex-matched healthy controls [31]. Notably, GSE55235 comprises synovial tissue primarily from non-hip (predominantly knee) joints; hip-specific, control-matched public datasets were not available at the time of analysis.

The dataset represents bulk synovial tissue and does not include layer annotations; therefore, subdivision into intima and subintima was not possible. For the present analysis, only OA and control samples were included, while RA samples were excluded as they were outside the scope of this study. Total RNA (3–5 µg) from each sample had been processed using the Affymetrix GeneChip^®^ one-cycle target labeling kit and hybridized onto Affymetrix arrays, with scanning performed on the GeneChip Scanner 3000 platform (Affymetrix, Santa Clara, CA, USA).

Raw data were analyzed using GEO2R, the built-in NCBI tool for differential expression (National Center for Biotechnology Information, Bethesda, MD, USA), which applies the limma R package (version 3.28.14; R Foundation for Statistical Computing, Vienna, Austria) with vooma precision weights and quantile normalization. Multiple testing correction was performed using the Benjamini–Hochberg false discovery rate (FDR) method. Genes were considered differentially expressed if they satisfied the following thresholds: |log_2_(fold change)| > 1 and adjusted *p* < 0.01. Genes with log_2_FC ≥ 1 were classified as upregulated, and those with log_2_FC ≤ −1 as downregulated. Accordingly, these public data were used as an orthogonal, joint-agnostic comparator to assess whether the directionality of IL6, TNFRSF1A, and TGFB1 expression observed in hip synovium is consistent at the tissue level, and should not be interpreted as a hip-specific (homologous) validation.

Visualization of differentially expressed genes was performed with a volcano plot, generated from the GEO2R output and refined for presentation using Adobe Photoshop, version 21.0.2 (Adobe, San Jose, CA, USA).

### 2.6. Statistical Analysis

All analyses were performed in GraphPad Prism (version 10.6.0; GraphPad Software, San Diego, CA, USA). Quantitative results are reported as mean ± standard deviation, and figures display group means with SD and individual data points where space allows. We compared four groups defined by compartment and disease status: CTRL-int, CTRL-sub, HOA-int, and HOA-sub. The prespecified contrasts were: (i) within-group int vs. sub in CTRL and in HOA, and (ii) between-group (HOA vs. CTRL) within each compartment (int and sub).

Primary analyses used two complementary approaches. When variability was comparable across groups and model assumptions were acceptable, we applied a two-way ANOVA with the factors compartment (int vs. sub) and disease status (HOA vs. CTRL), followed by Tukey’s multiple-comparisons test for the planned contrasts. When variability differed between groups or sample sizes were unbalanced, such as for TNFR1, we used a variance-robust framework: Brown–Forsythe one-way ANOVA across the four groups, followed by Dunnett’s T3 post hoc test for pairwise comparisons. For all post hoc procedures, adjusted *p*-values are reported.

For baseline demographic and clinical variables (Table 1), continuous data were compared among three independent groups: Controls, HOA Krenn 0–2, and HOA Krenn ≥ 3, using the Kruskal–Wallis test, while categorical variables (sex) were analyzed with the χ^2^ test. Clinical scores available only for HOA patients (HHS, VAS, and WOMAC) were presented descriptively without *p*-values. A *p*-value less than 0.05 was considered statistically significant. Significance levels are indicated in the Results section and figures as follows: *p* < 0.05 (*), *p* < 0.01 (**), *p* < 0.001 (***), and *p* < 0.0001 (****). Where applicable, we also report the exact adjusted *p*-values from Tukey or Dunnett’s T3.

## 3. Results

The protein expression patterns of tumor necrosis factor receptor 1 (TNFR1/CD120a), interleukin-6 (IL-6), and transforming growth factor-beta 1 (TGF-β1) were evaluated in synovial tissue specimens obtained from patients with hip osteoarthritis (HOA) and from control (CTRL) subjects without osteoarthritis. Immunofluorescence staining and quantitative analysis were performed, and the expression of each protein was assessed separately in the intima (int) and subintima (sub) compartments of the synovial membrane. Quantification was performed using morphology-based compartment masks as defined in Methods (Figure 1).

### 3.1. Hematoxylin and Eosin (H&E) Staining of the Synovial Membrane in Patients with Hip Osteoarthritis

**Figure 2:**
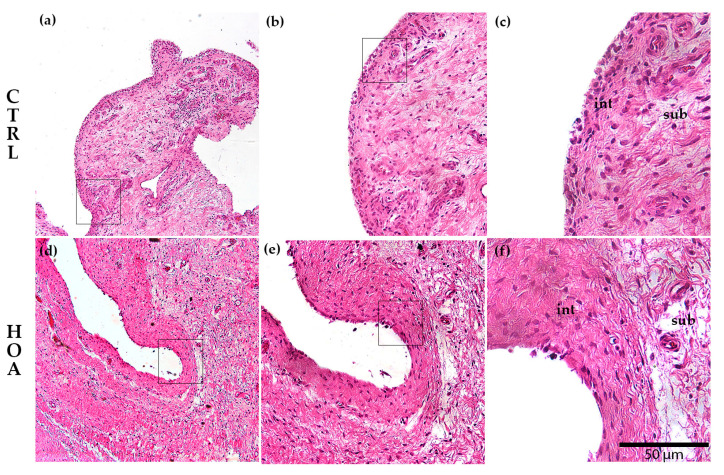
Histological comparison between control (CTRL) and hip osteoarthritis (HOA) synovium. Representative hematoxylin and eosin (H&E)-stained sections of synovial tissue are shown for CTRL (**a**–**c**) and HOA (**d**–**f**). The synovial intima (int), the continuous surface lining of synoviocytes, is thin in CTRL and hyperplastic in HOA. At the same time, the subintima (sub), the underlying fibro-areolar stroma, shows minimal infiltrate in CTRL and dense mononuclear inflammation with stromal activation in HOA. Images (**a**,**d**) were captured at 10× magnification, (**b**,**e**) at 20×, and (**c**,**f**) at 40×; scale bar = 50 μm; int and sub regions are indicated on the high-magnification panels (**c**,**f**).

Histological examination of H&E-stained synovial tissue revealed clear differences between CTRL and HOA samples (Figure 2). In the CTRL group, the synovial membrane exhibited a thin intimal lining composed of only a few cell layers, with sparse stromal activation and minimal inflammatory cell infiltration. By contrast, HOA samples demonstrated hallmark features of synovitis, including marked hyperplasia of the synovial lining, expansion and activation of stromal cells, and dense infiltration of mononuclear inflammatory cells within the sublining. These pathological alterations reflect both immune activation and structural remodeling of the synovial tissue in HOA, consistent with advanced disease morphology.

### 3.2. TNFR1 Is Predominantly Upregulated in the Intima of Hip Osteoarthritis Synovium

**Figure 3:**
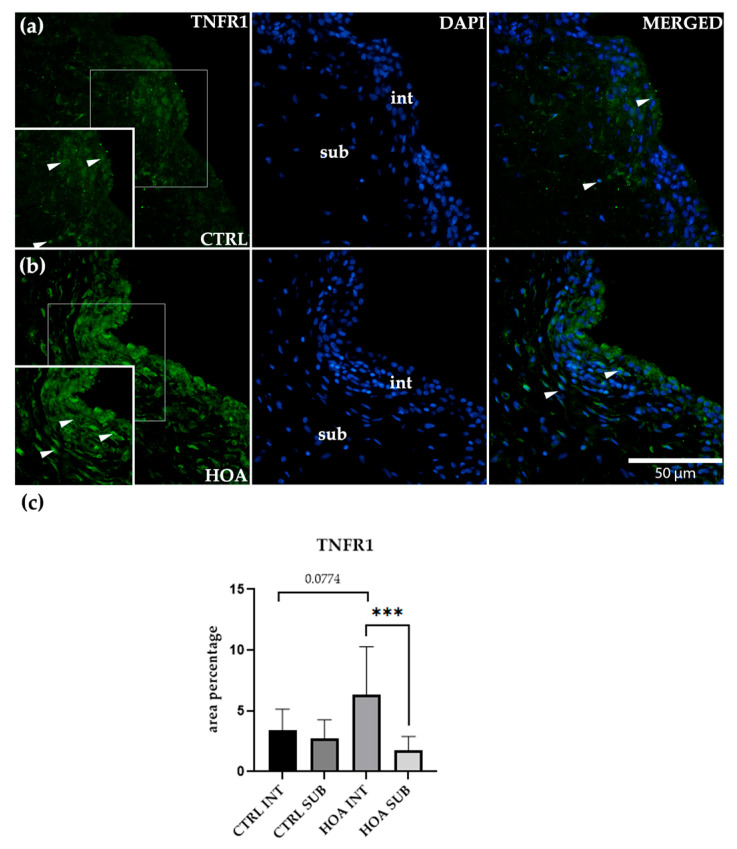
Immunofluorescence staining of tumor necrosis factor receptor 1 (TNFR1/CD120a) in control (CTRL) and hip osteoarthritis (HOA) synovial tissue (**a**,**b**). Sections were stained for TNFR1 (green) and counterstained with 4′,6-diamidino-2-phenylindole (DAPI, blue) to visualize nuclei. In the DAPI channel, the intima (int) and subintima (sub) are visible. Merged images show TNFR1 localization relative to nuclear staining. Arrows indicate TNFR1-positive cells within the synovial lining layer. Quantification of TNFR1 immunoreactivity in INT and SUB is shown in (**c**). Magnification: ×40; scale bar = 50 μm. Bars show mean ± standard deviation (SD) with individual data points. Owing to variance heterogeneity and unequal sample sizes, data were analyzed using Brown–Forsythe one-way ANOVA across CTRL-int, CTRL-sub, HOA-int, and HOA-sub, followed by Dunnett’s T3 multiple comparisons: *p* < 0.001 (***), *p* = 0.0774.

Immunofluorescence staining demonstrated that TNFR1 was detectable in both the intimal (int) and subintimal (sub) compartments of synovial tissue in CTRL and HOA samples (Figure 3a,b). In CTRL synovium, TNFR1 labeling was weak to moderate, with scattered positive cells along the INT and a low, diffuse signal in the SUB. Quantitatively, CTRL-int and CTRL-sub did not differ, indicating that under physiological conditions, TNFR1 distribution is relatively uniform across compartments (Figure 3c).

In the HOA synovium, a distinct compartmental pattern was evident. The int displayed a dense, band-like layer of TNFR1-positive cells with accentuated cytoplasmic staining along the luminal surface. In contrast, the sub showed weaker, discontinuous labeling without the pronounced linear profile seen in the int. Because variability differed between groups and sample sizes were not fully balanced, we analyzed TNFR1 using a variance-robust framework: Brown–Forsythe one-way ANOVA across the four groups (CTRL-int, CTRL-sub, HOA-int, HOA-sub) followed by Dunnett’s T3 post hoc testing for pairwise contrasts. This analysis confirmed a significant within-HOA compartment effect, with HOA-INT greater than HOA-SUB *p* < 0.001 (***). The between-group comparison for the intimal compartment, CTRL-int vs. HOA-int, showed a trend toward higher values in HOA but did not reach significance (*p* = 0.0774). Comparisons involving the subintimal compartment, including CTRL-sub vs. HOA-sub, were not significant. 

These findings refine the interpretation of TNFR1 behavior in HOA in light of variance heterogeneity. Rather than a uniform absolute increase across all groups, the dominant signal reflects a compartment-specific redistribution toward the synovial lining. The larger dispersion observed in HOA-int is consistent with biological heterogeneity typical of advanced synovitis, where variable degrees of lining hyperplasia, immune-cell infiltration, and stromal activation broaden the spread of values. Importantly, despite this dispersion, the interaction between HOA and TNFR1 remains robust at *p* < 0.001 (***), underscoring the lining as the principal site of TNFR1 upregulation in disease.

Taken together, the variance-robust statistics and the characteristic band-like INT staining pattern converge on the exact inference: TNFR1 upregulation in HOA is concentrated at the intimal surface, while the subintima remains unchanged mainly relative to controls. This intima-predominant signature supports a model of TNFR1-driven inflammatory signaling localized to the synovial lining (Figure 3c).

### 3.3. IL-6 Is Significantly Upregulated in the Intima of Hip Osteoarthritis Synovium

**Figure 4:**
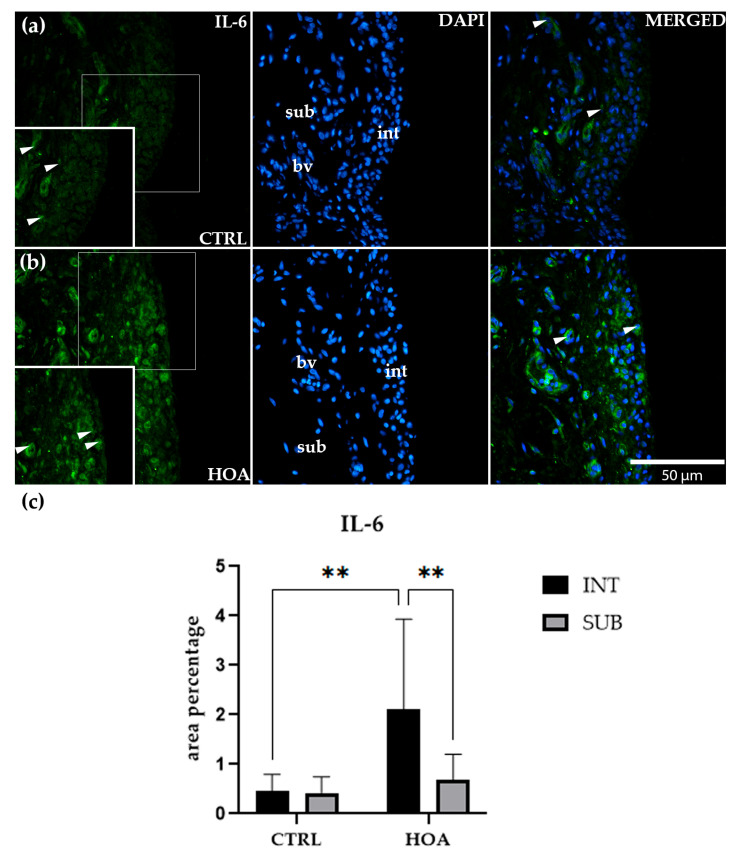
Immunofluorescence staining of interleukin-6 (IL-6) in control (CTRL) and hip osteoarthritis (HOA) synovial tissue (**a**,**b**). Sections were stained for IL-6 (green) and counterstained with 4′,6-diamidino-2-phenylindole (DAPI, blue) to visualize nuclei. In the DAPI channel, the intima (int), subintima (sub), and blood vessels (bv) can be distinguished. Merged images demonstrate IL-6 distribution relative to nuclear staining and synovial substructures. Arrows indicate IL-6-positive cells within the synovial lining layer. Quantification of IL-6 immunoreactivity in the int and sub compartments is presented in (**c**). Magnification: ×40; scale bar = 50 µm. Data are expressed as mean ± SD and analyzed by two-way ANOVA followed by Tukey’s multiple comparison test. *p*-value notation: *p* < 0.01 (**).

Immunofluorescence staining revealed that IL-6 was present in both the int and sub compartments of synovial tissue in CTRL and HOA samples (Figure 4a,b). In CTRL synovium (Figure 4a), IL-6 staining appeared weak and discontinuous, with only scattered positively stained cells visible within the int. Notably, IL-6–positive endothelial cells were also observed within the subintimal blood vessels (bv) of both CTRL and HOA samples, indicating vascular involvement as an additional source of signal. In HOA tissue, IL-6 immunoreactivity was not restricted to vascular endothelium/perivascular cells but was also present throughout the intimal lining and the surrounding subintimal stroma, consistent with a broader tissue distribution (Figure 4a,b). The sub-compartment showed similarly faint and diffuse labeling, without a clear compartmental pattern. Quantitative assessment confirmed that there was no significant difference between int and sub in CTRL tissue, indicating that IL-6 expression under physiological conditions is minimal and uniformly distributed across both compartments (Figure 4c).

In contrast, HOA samples (Figure 4b) displayed a more distinct staining profile. The int was characterized by a dense, continuous band of IL-6-positive cells, with a strong cytoplasmic signal outlining the synovial lining. This marked accumulation of staining sharply contrasted with the weaker and more diffuse signal observed in the adjacent sub. Quantification supported these observations, showing that IL-6 expression in the HOA int was significantly higher than in the CTRL int (*p* < 0.01). Furthermore, within the HOA group, the int showed significantly greater staining compared with the HOA sub (*p* < 0.01), confirming a compartment-specific increase in IL-6 restricted to the lining layer (Figure 4c).

Interestingly, IL-6 expression in the sub-compartment did not differ between CTRL and HOA samples. Both groups displayed only modest, low-level staining in this region, and no statistically significant differences were observed. Similarly, there was no difference between int and sub compartments within CTRL samples, further supporting that IL-6 is not differentially expressed under normal conditions. Thus, the compartmental differences observed were unique to HOA and driven explicitly by changes in the int.

Taken together, these findings demonstrate that IL-6 upregulation in HOA is highly compartmentalized, being concentrated within the synovial int while the sub remains unchanged. The striking contrast between weak, patchy staining in CTRL tissue and the dense, continuous IL-6 signal in OA int (Figure 4a,b) highlights the role of IL-6 as a localized mediator of synovial inflammation. These results suggest that the int acts as the primary site of IL-6–driven inflammatory activity in HOA, while the sub-compartment does not appear to contribute substantially to this process (Figure 4c).

### 3.4. TGF-β1 Intimal Expression Is Reduced in Hip Osteoarthritis Compared with Controls

**Figure 5:**
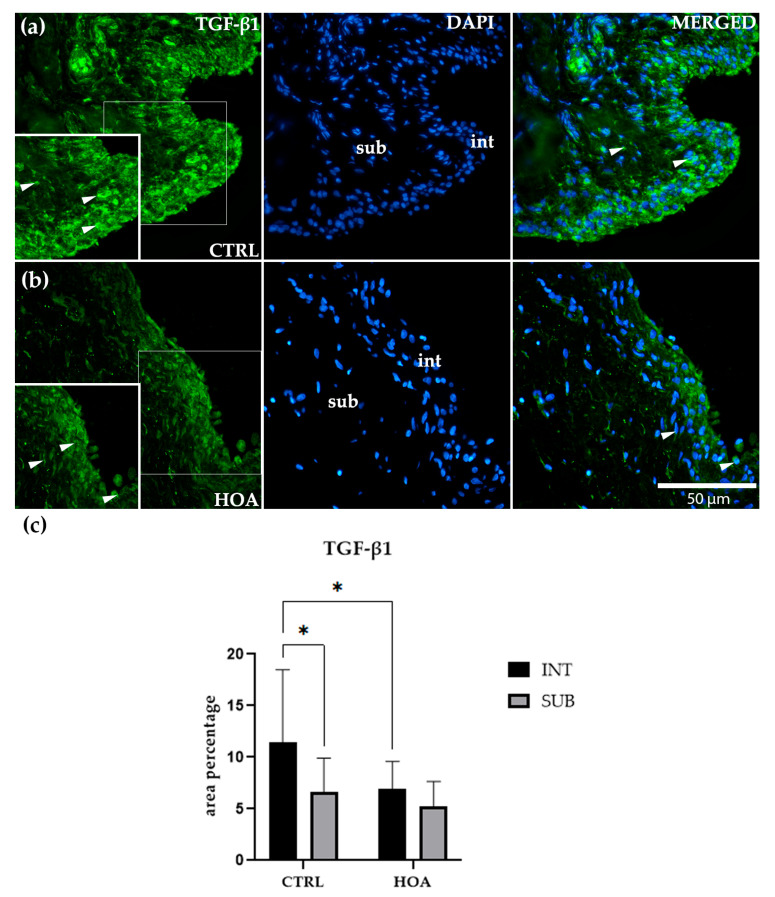
Immunofluorescence staining of transforming growth factor-beta 1 (TGF-β1) in control (CTRL) and hip osteoarthritis (HOA) synovial tissue (**a**,**b**). Sections were stained for TGF-β1 (green) and counterstained with 4′,6-diamidino-2-phenylindole (DAPI, blue) to visualize nuclei. In the DAPI channel, the intima (int) and subintima (sub) can be distinguished. Merged images demonstrate TGF-β1 expression in relation to nuclear staining. Arrows indicate TGF-β1-positive cells within the synovial lining layer. Quantification of TGF-β1 immunoreactivity in the int and sub compartments is presented in (**c**). Magnification: ×40; scale bar = 50 µm. Data are expressed as mean ± SD and analyzed by two-way ANOVA followed by Tukey’s multiple comparison test. *p*-value notation: *p* < 0.05 (*).

Immunofluorescence staining showed that TGF-β1 was detectable in both the int and sub compartments of synovial tissue in CTRL and HOA samples (Figure 5a,b). In CTRL synovium (Figure 5a), TGF-β1 staining was more prominent in the int compared with the sub. The int displayed a continuous band of positively stained cells along the lining, while the sub showed only a faint and scattered signal. Quantitative analysis confirmed that TGF-β1 expression in the CTRL int was significantly higher than in the CTRL sub (*p* < 0.05), suggesting that under physiological conditions, this growth factor is enriched in the lining layer of the synovium (Figure 5c).

In the HOA tissue (Figure 5b), TGF-β1 staining was present in both compartments but did not display the same clear separation seen in CTRL samples. The int remained positive, with scattered and moderate staining, but lacked the dense, continuous band observed for IL-6 or TNFR1. The sub demonstrated weak and diffuse staining, with no clear accumulation. Quantitative analysis indicated that TGF-β1 expression in the HOA int was significantly lower than in the CTRL int (*p* < 0.05), suggesting that HOA is associated with a reduction in TGF-β1 at the synovial lining. However, within HOA samples, there was no significant difference between the int and sub compartments, highlighting a loss of compartmental distinction for this cytokine in disease (Figure 5c).

No significant differences were observed between CTRL and HOA subcompartments, both of which showed low and diffuse TGF-β1 expression. Similarly, within the HOA group, expression levels were comparable between int and sub, confirming the absence of compartment-specific regulation in the diseased state.

Overall, these findings indicate that TGF-β1 displays a unique expression pattern compared with IL-6 and TNFR1. While CTRL samples showed higher TGF-β1 levels in the int compared with the sub, this compartmental enrichment was diminished in HOA. The visual impression of weaker and more diffuse staining in HOA compared with CTRL (Figure 5a,b) supports this interpretation, indicating that TGF-β1 plays a different role in synovial remodeling than the pro-inflammatory mediators IL-6 and TNFR1 (Figure 5c).

### 3.5. Transcriptomic Expression of IL-6, TNFR1, and TGF-β1 in Synovial Tissue

RNA sequencing (RNA-seq) data from the GSE55235 dataset (Rheumatoid arthritis and osteoarthritis: synovial tissues, Berlin cohort [31]) were analyzed to assess differential expression of interleukin-6 (IL6), Tumor necrosis factor receptor 1 (TNFRSF1A), and transforming growth factor-beta 1 (TGFB1) between osteoarthritis (OA) and healthy control (CTRL) synovial tissues. Genes with |log_2_(fold change)| > 1 and p < 0.01 were considered significantly differentially expressed.

**Figure 6:**
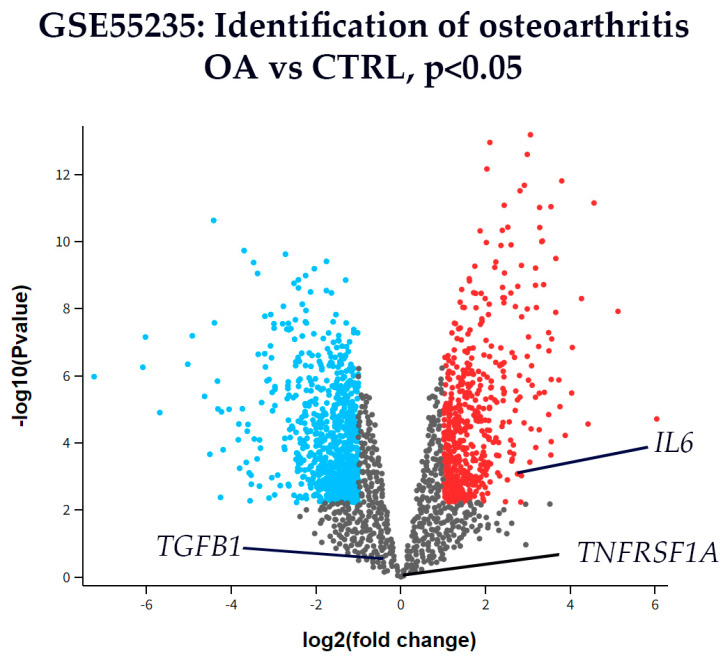
Volcano plot of differentially expressed genes in synovial tissue from osteoarthritis (OA) and control (CTRL) patients from dataset GSE55235. The x-axis represents the log_2_(fold change), and the y-axis shows the –log_10_(*p*-value). Genes with *p* < 0.01 (–log_10_(*p*) > 2) are shown in color: red for upregulated and blue for downregulated genes, while all others are shown in gray. This dataset represents bulk synovium and is knee-predominant (non-hip) with no layer annotations; therefore, the analysis reflects overall OA vs. CTRL directionality and is not layer-specific. Among the analyzed targets, *interleukin-6* (*IL6*) was significantly upregulated in OA compared to CTRL. *Tumor necrosis factor receptor 1* (*TNFRSF1A*) and *transforming growth factor-beta 1* (*TGFB1*) are also labeled for reference, but did not reach statistical significance. These transcriptomic results are used as orthogonal support to the hip immunofluorescence findings rather than as homologous, layer-resolved validation.

Among the three analyzed genes, *IL*-*6* was significantly upregulated in OA synovial tissue compared with CTRL, reaching the predefined threshold for both fold change and statistical significance (Figure 6). In contrast, *TNFRSF1A* and *TGFB1* did not show significant differential expression between OA and CTRL tissues, as their fold changes did not meet the cutoff values.

## 4. Discussion

The synovial membrane plays a central role in the pathogenesis of OA, acting as both a target and source of inflammatory mediators that drive joint degeneration and remodeling [32,33]. Cytokines such as IL-6, TNFR1, and TGF-β1 are key regulators of synovial biology, influencing processes including immune cell recruitment, extracellular matrix turnover, and tissue repair [9]. Although these molecules have been extensively studied in serum, synovial fluid, and animal models, their compartment-specific expression within human hip synovium remains less well characterized [34]. By focusing on primary HOA, we aimed to provide novel insights into how inflammatory and regulatory signals are distributed within distinct synovial compartments and how these patterns may contribute to disease progression.

In our study, TNFR1 immunoreactivity was predominantly enriched in the intimal lining of HOA synovium, with a robust within-disease compartment effect (int > sub), while subintimal levels remained comparable between HOA and CTRLs. The between-group intimal contrast (HOA int vs. CTRL int) showed a trend but did not reach significance after variance-robust testing, indicating that the dominant signal reflects compartmental redistribution rather than a uniform absolute increase. This compartment-specific elevation underscores the intima as the dominant site of TNFR1 activity in HOA.

Experimental models highlight the importance of this compartment. Arntz et al. demonstrated that TNFR1 signaling in synovial lining cells and reticuloendothelial tissues is crucial for sustaining experimental arthritis. Selective disruption of TNFR1 in lining cells markedly reduced joint inflammation, diminished IL-1β and IL-6 release, and protected cartilage integrity [35]. These results show that lining-cell TNFR1 is sufficient to drive pathology, consistent with our observation of selective intimal upregulation in human HOA.

Human data provide parallel support. Semenistaja et al. profiled synovial tissue from patients with late-stage OA and identified distinct inflammatory phenotypes. High-grade synovitis was characterized by lining hyperplasia, immune infiltration, enhanced NF-κB activity, and elevated TNF-α expression [36]. Such features mirror our findings of intimal TNFR1 enrichment, suggesting that TNFR1-dependent pathways in the lining are closely linked to the inflammatory and symptomatic burden of advanced HOA. The greater dispersion observed in HOA int is also consistent with biological heterogeneity typical of advanced synovitis.

Synovium–cartilage interactions may further amplify this effect. Chou et al. showed that synovial cells communicate extensively with chondrocytes, and that TNF-α is a major driver of catabolic changes in cartilage [37]. Complementing this, Webb et al. reported that OA synovial fluid and supernatants upregulate p55 TNFR1 on human articular chondrocytes, increasing their susceptibility to TNF-α-induced proteoglycan loss [38]. Together, these findings suggest that intimal TNFR1 upregulation may prime cartilage for catabolic damage via cytokine-mediated cross-talk.

Finally, therapeutic opportunities targeting this pathway are emerging. Li et al. reviewed recent advances in TNFR1-selective inhibitors, including Atrosimab and SAR441566, which block deleterious TNFR1 signaling while sparing the protective and regenerative functions of TNFR2 [11]. Our finding of compartment-specific TNFR1 enrichment in the intima provides a strong rationale for such strategies, which could attenuate synovial inflammation and cartilage vulnerability without inducing broad immunosuppression.

Beyond expression patterns, TNFR1 function may be further enhanced in OA via post-translational modification. Yu et al. showed that α-1,3 fucosylation of TNFR1 in osteoarthritic cartilage increases its binding affinity to TNF-α, amplifying NF-κB and MAPK signaling, and promoting chondrocyte apoptosis and extracellular matrix degradation. This mechanistic insight complements our observation of elevated intimal TNFR1 expression by showing that cartilage cells become more sensitive to TNF-mediated catabolism [39].

Taken together, our results and other recent studies support a model in which HOA is characterized by an intima-predominant redistribution of TNFR1 within diseased synovium, amplifying synovial inflammation and sensitizing cartilage to damage [35,36,37,38,39]. From a translational perspective, both lining-cell TNFR1 signaling and cartilage susceptibility to TNFR1 activation emerge as critical targets for interventions aiming to disrupt the TNF/NF-κB axis in HOA [11].

In our study, IL-6 immunoreactivity was markedly enriched in the intimal lining of HOA synovium, while the subintima showed no significant change compared to CTRLs. This compartment-specific pattern identifies the synovial intima as the dominant niche of IL-6 activity in HOA.

These findings are consistent with human biopsy data. Benito et al. demonstrated that early OA synovium exhibits pronounced infiltration of CD4⁺ T cells and macrophages, coupled with overproduction of IL-6 and other pro-inflammatory cytokines. Nuclear localization of NF-κB subunits was also observed, indicating active transcriptional upregulation of IL-6 in the lining layer [33]. This inflammatory profile parallels our observation of selective intimal IL-6 enrichment, supporting the idea that the lining is a critical driver of inflammatory signaling in OA.

Single-cell transcriptomic studies provide further mechanistic insight. Chou et al. identified distinct HLA-DRA⁺ inflammatory macrophages and dendritic cells in OA synovium as major sources of IL-6 and IL-1β, with expression concentrated in the intimal compartment [37]. Similarly, Zhang et al. highlighted fibroblast-like synoviocytes as central mediators of IL-6–driven fibrosis and inflammation in OA synovium [40]. These results reinforce that intimal fibroblasts and immune cells together create a cytokine-rich microenvironment consistent with our immunofluorescence findings.

Functionally, IL-6 has been directly linked to both structural damage and pain. Stannus et al. showed that higher serum IL-6 levels predicted greater radiographic progression of knee OA over five years [41]. Sullivan et al. further demonstrated a positive correlation between synovial fluid IL-6 concentrations and pain scores after ACL injury and reconstruction, underscoring the role of IL-6 in driving nociception. [42]. Together, these data explain why the intimal enrichment of IL-6 observed in our study is not only a marker of local inflammation but also likely contributes to clinical symptoms in HOA.

Finally, IL-6 is closely tied to angiogenesis and tissue remodeling. Lambert et al. reported that IL-6 production in inflamed OA synovium was coupled with VEGF upregulation and enhanced vascular density [43]. In our cohort, strong intimal IL-6 staining adjacent to the synovial surface may similarly reflect an angiogenic and catabolic microenvironment that perpetuates OA pathology.

Taken together, our results align with and extend current evidence by showing that IL-6 upregulation in HOA is highly compartmentalized: concentrated within the intimal lining, while subintimal levels remain unchanged. This pattern mirrors the localization of its main cellular producers and provides a mechanistic link to both inflammation and pain. These insights suggest that therapeutic strategies targeting the IL-6/JAK/STAT axis should consider compartment-specific effects, with the intima emerging as the primary site of pathogenic IL-6 activity in HOA.

In our study, TGF-β1 immunoreactivity was stronger in the intimal compartment of CTRL synovium but diminished in HOA, thereby eliminating the normal intima–subintima gradient. This contrasts with IL-6, where HOA markedly increased intimal expression. Importantly, TGF-β1 expression in the subintima remained unchanged between groups, suggesting that TGF-β1 regulation in HOA is compartment-specific rather than globally altered.

Several studies highlight a different picture in other joint compartments. Muratovic et al. demonstrated that active TGF-β1 levels are elevated in subchondral bone beneath degenerated cartilage, correlating with deteriorated bone quality and disease severity [44]. This indicates that while bone and cartilage compartments experience heightened TGF-β1 activity, our HOA synovium shows a relative loss at the intimal surface, emphasizing spatial heterogeneity of TGF-β signaling within the joint.

Mechanistic studies provide one explanation for this discrepancy. Ciregia et al. showed that αVβ6 integrin is upregulated in OA synovial fibroblasts, where it activates latent TGF-β1 and drives α-SMA expression and fibrosis. This integrin-mediated activation could sustain downstream TGF-β1 activity even when overall protein levels detected by immunofluorescence appear reduced [45]. Such findings align with our observation that intimal protein levels are decreased in OA, while disease-associated activation persists through post-translational mechanisms.

At the cartilage level, Thielen et al. demonstrated that OA chondrocytes exposed to high TGF-β concentrations undergo a signaling shift from protective SMAD2/3 toward detrimental SMAD1/5/9 activation. This promoted hypertrophic markers such as RUNX2 and COL10A1, contributing to cartilage degeneration [46]. In light of our data, reduced intimal TGF-β1 could reflect a loss of protective SMAD2/3-mediated signaling, leaving cartilage vulnerable to this maladaptive SMAD1/5/9-driven response.

Earlier work by Scharstuhl et al. already showed that persistent high TGF-β activity induces fibrosis and osteophyte formation in joint tissues [47]. Coupled with newer data, these findings suggest that OA is not simply a matter of “increased or decreased” TGF-β1, but of altered compartmental distribution and signaling bias.

Finally, evidence from inflammatory arthritis supports this compartment-specific view. Pinto-Tasende et al. reported that synovial TGF-β1 immunofluorescence reactivity was significantly higher in patients with early psoriatic and rheumatoid arthritis, where it predicted the later need for biologic therapy [48]. This stands in contrast to our observation of diminished intimal staining in HOA, suggesting that TGF-β1 behaves differently depending on disease type and stage: elevated early in inflammatory arthritis, but downregulated at the intimal surface in chronic, late-stage OA.

Thus, our study highlights that synovial intimal TGF-β1 expression is reduced in HOA, diverging from reports of increased activity in bone, cartilage, and inflammatory arthritis. Taken together, in our study, these results underscore the compartment-specific dysregulation of TGF-β1: diminished intimal expression, persistent integrin-mediated activation in fibroblasts [45], excessive subchondral bone activity is linked to degeneration [44], and early overexpression predicting aggressive disease in PsA/RA [48]. Therapeutically, this emphasizes the need for pathway and compartment-specific modulation, for example, selectively inhibiting detrimental SMAD1/5/9 signaling while preserving protective SMAD2/3 activity.

We complemented our protein-level findings with differential expression analysis using GEO GSE55235. Importantly, this dataset represents non-hip (predominantly knee) synovium analyzed on an Affymetrix microarray; thus, it serves as an orthogonal reference rather than a hip-specific (homologous) validation. Despite possible joint-site differences, the observed IL6 upregulation with unchanged TNFRSF1A and TGFB1 trends aligns directionally with our results and supports their robustness. 

This study has several limitations. The sample size was relatively small (19 HOA and 10 control subjects), which may limit statistical power; therefore, larger multicenter studies are needed to validate these findings. The transcriptomic dataset (GSE55235) used for comparison included mainly knee synovial tissue, serving only as an orthogonal reference for cytokine directionality rather than a hip-specific validation. Moreover, the study was descriptive and correlational, lacking functional experiments to determine causality. Because viable human hip synovium for molecular manipulation cannot be ethically or technically obtained, such validation will require in vitro or in vivo models. Future validation will require larger, multicenter cohorts with harmonized protocols, prospective biobanking, and pre-specified power calculations to confirm these compartment-specific findings and enable more granular stratification.

## 5. Conclusions

This study demonstrates compartment-specific cytokine alterations in the synovial membrane of hip osteoarthritis. Within HOA, TNFR1 and IL-6 were enriched at the synovial intima; for TNFR1 the increase was significant for the intima versus subintima within HOA, while the intimal HOA versus CTRL contrast showed a non-significant trend. IL-6 was significantly upregulated in the intimal lining, whereas TGF-β1 expression was reduced, leading to the loss of the normal intima–subintima gradient. Collectively, these findings indicate an intima-predominant redistribution of inflammatory signaling in HOA, identifying the synovial lining as a key site of pathogenic activity and clarifying the molecular pathology underlying disease progression.

## Figures and Tables

**Table 1:** Clinical, radiological, and pathohistological characteristics of the examined groups.

Characteristic	Controls (*n* = 10)	HOA Krenn 0–2 (*n* = 10)	HOA Krenn ≥ 3 (*n* = 9)	*p*-Value
Age (years), median (IQR)	74 (73.6–76.1)	73 (63.7–75.9)	73 (66–78)	0.854
Sex (Male/Female)	6/4	6/4	5/4	0.732
BMI (kg/m^2^), median (IQR)	25.9 (24.0–26.6)	24.7 (23.3–25.8)	26.7 (25.5–29.4)	0.054
K–L grade, median (IQR)	0.5 (0–1)	2 (2–2)	4 (3–4)	<0.0001
Krenn synovitis score, median (IQR)	0 (0–0)	6.4 (5.6–9.0)	9 (7–9)	<0.0001
Harris Hip Score (HHS)	–	48.7 (43.5–56.8)	41 (33.5–49.6)	*
VAS (0–10)	–	6 (4.6–6.8)	6 (5–7)	*
WOMAC	–	46.2 (40.2–56.4)	47.3 (36.1–55.3)	*

Abbreviations: IQR = interquartile range; HOA = hip osteoarthritis; BMI = body mass index; K–L grade = Kellgren–Lawrence grading scale; HHS = Harris Hip Score; VAS = visual analogue scale; WOMAC = Western Ontario and McMaster Universities Osteoarthritis Index. Statistical analysis: *p* < 0.05 was considered statistically significant. Continuous variables were compared among the three groups (Controls, HOA Krenn 0–2, HOA Krenn ≥ 3) using the Kruskal–Wallis test, and categorical variables (sex) were compared using the χ^2^ test. * Variables not measured in controls; values reported only for HOA subgroups and presented descriptively without *p*-values.

**Table 2:** Antibodies used for immunofluorescence.

Antibody Type	Antibody	Host	Dilution	Source
Primary	Anti-TNFR1/CD120a (21574-1-AP)	Rabbit	1:300	Proteintech, Rosemont, IL, USA
	Anti-IL-6 (66146-1-Ig)	Mouse	1:200	
	Anti-TGF-β1 (21898-1-AP)	Rabbit	1:300	
Secondary	Alexa Fluor^®^ 488 Anti-Rabbit IgG (711-545-152) Alexa Fluor^®^ 488 AffiniPure^®^ Donkey Anti-Mouse IgG (H+L) (715-545-150)	Donkey	1:300	Jackson ImmunoResearch Laboratories, Inc. (West Grove, PA, USA)

## Data Availability

The original contributions presented in this study are included in the article. Further inquiries can be directed to the corresponding author(s).

## Abbreviations

The following abbreviations are used in this manuscript:

TNFR1	Tumor necrosis factor receptor 1
IL-6	Interleukin-6
OA	Osteoarthritis
ANOVA	Analysis of variance
HOA	Hip osteoarthritis
TNF	Tumor necrosis factor
NF-κB	Nuclear factor kappa-light-chain-enhancer of activated B cells
RA	Rheumatoid arthritis
JAK	Janus kinase
STAT	Signal transducer and activator of transcription
ECM	Extracellular matrix
SMAD2/3	Mothers against decapentaplegic homologs 2 and 3 (canonical TGF-β pathway)
MAPK	Mitogen-activated protein kinase
SMAD1/5/9	Mothers against decapentaplegic homologs 1, 5, and 9 (TGF-β pathway)
CCP	Cyclic citrullinated peptide
RF	Rheumatoid factor
HHS	Harris Hip Score
WOMAC	Western Ontario and McMaster Universities Osteoarthritis Index
VAS	Visual analogue scale
IQR	Interquartile range
BMI	Body mass index
BX	Olympus BX brightfield microscope
PBS	Phosphate-buffered saline
DAPI	4′,6-diamidino-2-phenylindole
BX61	Olympus BX61 fluorescence microscope
NIH	National Institutes of Health
SD	Standard deviation
GEO	Gene Expression Omnibus
NCBI	National Center for Biotechnology Information
GSE55235	GEO dataset series 55235
RNA	Ribonucleic acid
GEO2R	Gene Expression Omnibus online analysis tool 2R
FDR	False discovery rate
CTRL	Control
SAR44156	TNFR1-selective inhibitor (drug candidate)
TNFR2	Tumor necrosis factor receptor 2
CD4	Cluster of differentiation 4
HLA-DRA	Human leukocyte antigen DR alpha
ACL	Anterior cruciate ligament
VEGF	Vascular endothelial growth factor
α-SMA	Alpha-smooth muscle actin
RUNX2	Runt-related transcription factor 2
COL10A1	Collagen type X alpha 1 chain
